# Electrical stimulation of the cerebellum facilitates automatic but not controlled word retrieval

**DOI:** 10.1007/s00429-023-02712-0

**Published:** 2023-10-02

**Authors:** Dominika Petríková, Martin Marko, Rastislav Rovný, Igor Riečanský

**Affiliations:** 1 Department of Behavioural Neuroscience, Centre of Experimental Medicine, Slovak Academy of Sciences, Sienkiewiczova 1, 81371 Bratislava, Slovakia; 2 Department of Applied Informatics, Faculty of Mathematics, Physics and Informatics, Comenius University in Bratislava, Bratislava, Slovakia; 3 Department of Psychiatry, Faculty of Medicine, Slovak Medical University in Bratislava, Bratislava, Slovakia

**Keywords:** Lexical-semantic retrieval, Semantic memory, Cerebellum, Non-invasive brain stimulation (NIBS), Cognitive control, Verbal fluency

## Abstract

Recent research has indicated that the cerebellum is engaged in language functions, yet the role of the cerebellum in lexical-semantic memory is poorly understood. In a double-blind randomized controlled experiment, we therefore targeted the cerebellum by transcranial direct current stimulation (tDCS) to assess and compare the contribution of the cerebellar processing to automatic and controlled retrieval of words in healthy adults (n = 136). Anodal cerebellar tDCS facilitated retrieval of semantically related words in free-associative chains, which was not due to a non-specific acceleration of processing speed. The stimulation had no influence on controlled word retrieval that employed inhibition or switching. The effect of cathodal tDCS was opposite to the anodal stimulation, but statistically non-significant. Our data show that the cerebellum is engaged extracting associative information from the system of semantic representations, established and strengthened/automated by learning, and indicates a domain-general role of this structure in automation of behavior, cognition and language.

**Supplementary Information:**

The online version contains supplementary material available at 10.1007/s00429-023-02712-0.

## Introduction

Although the current views highlight the role of the cerebral cortex in semantic representation and control (Lambon Ralph et al. 2017), several lines of evidence indicate that semantic cognition is also supported by the activity of the cerebellum. For instance, patients with cerebellar lesions show deficits in semantic (category) verbal fluency task, which requires generating exemplars from a specific semantic category (Molinari and Leggio 2016). Activation of the cerebellum in this task (Gurd et al. 2002; Nagels et al. 2012; Li et al. 2017; Rodríguez-Aranda et al. 2020), as well as other tasks in which semantically related words have to be delivered, has been also documented in functional neuroimaging studies (Mariën and Borgatti 2018; Stoodley and Schmahmann 2016). In addition, it has been reported that semantic fluency is positively related to cerebellar grey matter volume (Rodríguez-Aranda et al. 2016). Furthermore, semantic dementia, a disorder characterized by a relatively selective degradation of semantic memory, is associated with reduced cerebellar activity and gray matter volume (Chen et al. 2019). These and other related findings indicate that the cerebellum contributes to retrieval of semantically related representations, which has been interpreted from the perspective of the cerebellum’s role in generating context-based predictions (Lesage et al. 2012, 2017; Moberget et al. 2014; Argyropoulos 2016; Sokolov et al. 2017; Gatti et al. 2021a).

Moreover, numerous studies have shown that the cerebellum is involved in executive control functions (Bellebaum and Daum 2007; Hoche et al. 2018) and functional neuroimaging studies have indicated the cerebellum is also engaged in the executive control of semantic memory (Canini et al. 2016; Hallam et al. 2016; Gonzalez Alam et al. 2019). Interestingly, when compared with category fluency, performance of patients with cerebellar damage is usually more heavily impaired in phonemic (letter) verbal fluency task, which requires individuals to produce words that begin with a specific letter. Compared with category fluency, the lexical search in phonemic fluency is less usual/natural and seems to involve executive suppression of automatic (habitual, pre-potent) semantic associates (Marko et al. 2023).

Thus, it is currently unclear whether, or to which extent, the cerebellum contributes to specific lexical-semantic processes—(i) automatic retrieval, i.e. the process of a bottom-up activation of semantic representations triggered by environmental cues or spontaneous thought, and (ii) the retrieval’s executive control, i.e. a top-down manipulation of semantic processing employed if the outputs from automatic retrieval are inappropriate for the current task, respectively. We addressed this question by adopting the associative chain test (ACT, Marko et al. 2019), in which automatic (free association) and controlled (inhibition and switching) retrieval can be effectively disentangled. In ACT, individuals generate sequences of words based on specific rules—associative (production of semantically related words), dissociative (production of semantically unrelated words), or alternating (production of semantically related and unrelated words in alternation). To manipulate cerebellar activity, we employed transcranial direct current stimulation (tDCS), which has been shown to modulate the activity of the cerebellum and performance in a number of cognitive tasks, including those engaging lexical-semantic processes (for review see van Dun et al. 2017). The rationale for this approach was that a modulatory effect of the cerebellar stimulation on ACT performance would reveal the relative contribution of the cerebellum’s activity to automatic as compared to controlled lexical-semantic retrieval. To assess the specificity of the tDCS effects, we also adopted two control tasks. One of these was a sentence completion task, in which semantic retrieval serves to predict occurrence of a future word, and following previous research, we expected that excitatory cerebellar tDCS would aid completing predictable rather than unpredictable sentences (D’Mello et al. 2017; Lesage et al. 2017; Rice et al. 2021). The second task was a simple choice reaction time task, to control for eventual effects of the stimulation on domain-general processing speed (Wong et al. 2021).

## Materials and methods

### Participants

Participants were healthy adults, native Slovak speakers, with no history of psychiatric or neurological disorder or current medication. The sample included 136 individuals who were randomly assigned into groups receiving either sham tDCS (*n* = 45), anodal (*n* = 45) or cathodal tDCS (*n* = 46). The sample size was based on our previous study, which revealed significant effects of tDCS on ACT performance (Marko and Riečanský 2021). Assuming effect of a similar size, this sample would achieve power larger than 0.80. Basic characteristics of the sample are provided in Table 1. The study was conducted in accordance with the Declaration of Helsinki (World Medical Association 2013) and approved by the institutional review board. All participants gave written informed consent and received a financial reward for their participation.

**Table 1:** Basic characteristics of the sample

	Anodal	Cathodal	Sham	Statistical analysis
Sample size	45	46	45	
Gender male/female	22/23	21/25	23/22	*χ*^*2*^(1) = 0.27, *p* = .872 ^b^
Age in years	23.2 (± 3.34)	25.3 (± 4.99)	22.6 (± 2.38)	*χ*^*2*^(2) = 6.66, *p* = .036 ^c^
Handedness score^a^	0.7 (± 0.55)	0.7 (± 0.58)	0.7 (± 0.43)	*χ*^*2*^(2) = 2.09, *p* = .353 ^c^

The values for age and handedness represent mean (± SD)

^a^Simplified Hand Preference Questionnaire (Bryden 1977)

^b^Chi-square test

^c^Kruskal-Wallis test

### Study design and procedure

**Fig. 1:**
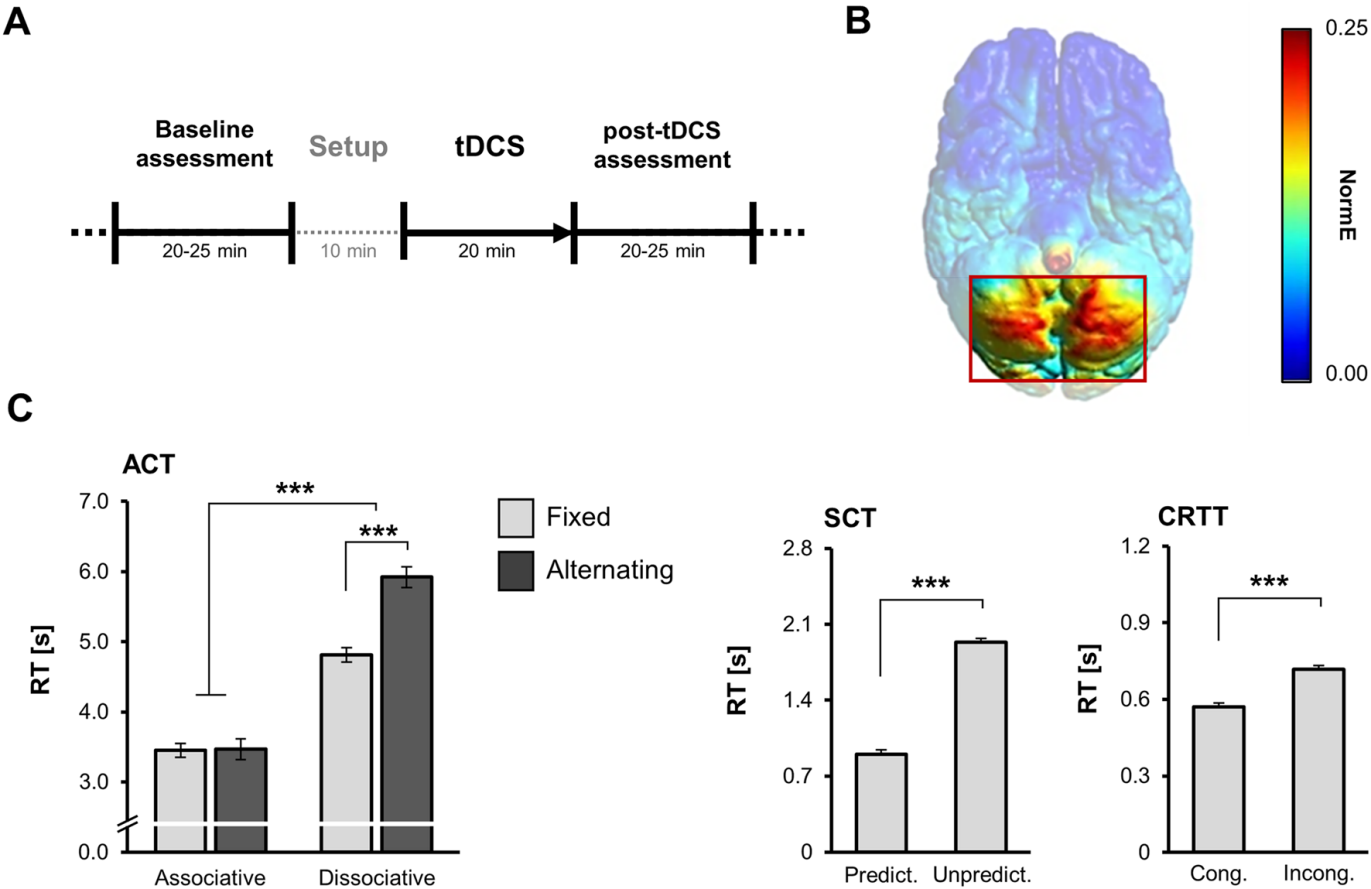
**A** Timeline of the experimental procedure. The cognitive tests (ACT, SCT, and CRTT, see main text) were administered before (baseline) and immediately after tDCS (post-tDCS). The order of administration of ACT and SCT was counterbalanced across participants, while CRTT was always administered at the end of the block. For each participant, the tasks were administered in the same order in both blocks. **B** Electric field intensity model of the cerebellar tissue polarization (SimNIBS version 3, (Thielscher et al. 2015). Due to software limitations of possible electrode montages, the model was calculated for a reference electrode placed on the right side of the neck. (C) Baseline performance in the cognitive tasks across the groups. Error bars: ± *SE*; *p < .05, **p < .01, ***p < .001

The study was a double-blind randomized controlled experiment with one between-subjects factor (*stimulation*: anodal, cathodal, sham) and one within-subjects factor (*block*: baseline, post-tDCS). The session started with a brief interview on medical history, followed by information about the procedures and administration of questionnaires. Next, baseline data from the cognitive tests were collected (20–25 min). Thereafter, tDCS montage was set up and participants answered additional questions (10 min). The stimulation period followed (20 min), during which no cognitive testing was carried out, and participants watched an emotionally neutral soundless video of natural sceneries. Immediately after tDCS, the cognitive assessment was repeated (post-tDCS block, 20–25 min). Thereafter, questionnaires were administered again including assessment of side effects of the stimulation. The basic timeline of the procedure is depicted in Fig. 1A.

### Transcranial direct current stimulation

A certified stimulator (DC-STIMULATOR PLUS, NeuroConn, Ilmenau, Germany) and rubber electrodes (5 × 7 cm^2^) were used. The stimulating electrode was centered at the midline 1–2 cm below the inion and the reference electrode was placed on the right arm in order to target the posterior cerebellum bilaterally (Ferrucci et al. 2015). Current intensity was 2 mA (0.057 mA/cm^2^) and impedance was kept below 10 kΩ. In the active conditions, the current was applied for 20 min with a 60 s ramp-up and ramp-down period. In the sham condition, the montage was identical, but the full-intensity current was delivered only for 40 s. The experimenter was not aware of whether real or sham stimulation was applied (double-blinding).

### Cognitive assessment

#### Associative chain test (ACT)

Lexical–semantic retrieval was assessed using ACT, described in a detail elsewhere (Marko et al. 2019). In short, each participant generated word chains according to specific rules starting with a stimulus word. There were three types of chains: associative, dissociative, and alternating. In the associative chain (Associative Fixed condition), each response had to be semantically related to the previous one (e.g., *“Music → Lyrics → Singer …”*). In the dissociative chain (Dissociative Fixed condition), each response had to be semantically unrelated to the previous one (e.g., *“Glasses ↛ Lizard ↛ Apple …”*). Finally, in the alternating chain, semantically related and unrelated words were delivered in alternation (e.g., *“School → Student ↛ Cake → Strawberry ↛ Train…”*), yielding the Associative Alternating and Dissociative Alternating conditions. The first two chains continued until participants delivered 20 responses, while the alternating chain lasted until a total of 40 responses were provided (i.e., 20 associations and 20 dissociations). Participants entered the words via keyboard, and they were instructed to keep fluent word production, retrieve only nouns, ignore grammatical or typing errors, and not repeat words within the same chain. Each response was assessed for response time (RT), i.e., the latency of entering the first letter of the word.

#### Sentence completion task (SCT)

In SCT, four words of a five-word sentence were presented sequentially (each displayed for 500 ms) and participants were requested to deliver (via keyboard) the last word to complete the sentence in a meaningful way. In total, there were 120 sentences presented in random order. The sentences had either predictable or unpredictable context (60 each), which was defined by cloze probability scores > 0.67 or < 0.33, respectively (Block and Baldwin 2010), estimated in a pilot study (for details, see Supplementary information). Completion RT was assessed as the latency of entering the first letter of the word.

#### Choice response time task (CRTT)

In CRTT, the stimuli were sequentially presented white or red arrows pointing in four possible directions (up, down, right, or left; Marko and Riečanský 2023). If a white arrow was displayed, the participants had to press the arrow key in the same direction as the arrow (congruent trials). If a red arrow was displayed, the task was to press the arrow key in the opposite direction (incongruent trials). The task consisted of three blocks of 80 trials (60 congruent, and 20 incongruent, in random order) with a pause of 5 s between the blocks. Each response was assessed for RT, i.e., the latency of pressing the key.

### Data processing and analysis

Data were processed in R Studio (RStudio Team 2020) and analyzed using R Statistical Software (R Core Team 2021). Prior to statistical analysis, for ACT, responses with large RTs (> 20 s) were removed alongside with responses from the block where participant provided less than half of the expected number of responses or produced words other than nouns. The remaining responses were then evaluated by two independent raters for accuracy (i.e., identifying responses that broke the rule: unrelated words in the associative conditions and related words in the dissociative conditions). Overall, 4.54% of responses were removed. For SCT, missing trials, non-sense responses, and responses not meeting the cloze probability criteria were removed (overall, 3.8% of trials). For CRTT, incorrect responses were removed (2.78% of the responses). For all tasks, RTs were winsorized (20% trimming, two-sided) separately for each participant, assessment block and condition. RT data were analyzed using linear mixed effect models (LMEM; R package lme4; Bates et al. 2015) to account for the measurements (individual RTs) nested within participant by estimating a random intercept for each participant (default unstructured covariance matrix). LMEMs were fitted using restricted maximum likelihood (REML) and *p*-values were derived with Satterthwaite approximation for degrees of freedom, as these were shown to produce optimal estimates even for smaller samples (Luke 2017). Post-hoc pairwise comparisons between the levels of each experimental factor were evaluated using Wald’s statistic and Satterthwaite approximation of degrees of freedom. The *p*-values of the post-hoc tests were corrected with Tukey’s honestly significant difference (HSD) adjustment to account for family-wise error rate (adjusted *p*-values are reported). Baseline (pre-tDCS) data were compared between the groups in each task to assess whether the groups differed in any measured parameter before the stimulation. This was done with LMEMs including a fixed between-subjects factor *stimulation* (sham, anodal, cathodal) for every condition in each task. To assess the effects of stimulation in all three tasks LMEMs were calculated including a fixed between-subjects factor *stimulation* (sham, anodal, cathodal), a fixed within-subjects factor *block* (baseline, post-tDCS) and a fixed within-subjects factor *condition* (in ACT: associative fixed, associative alternating, dissociative fixed, dissociative alternating; in SCT: predictable and unpredictable; and in CRTT: congruent and incongruent). In case the *stimulation x block x condition* interaction was significant, LMEMs were calculated for each condition separately. In addition, post-tDCS vs. baseline differences were estimated from the LMEM and compared pairwise between groups using Holm’s correction of *p*-values.

## Results

### Baseline performance

**Fig. 2:**
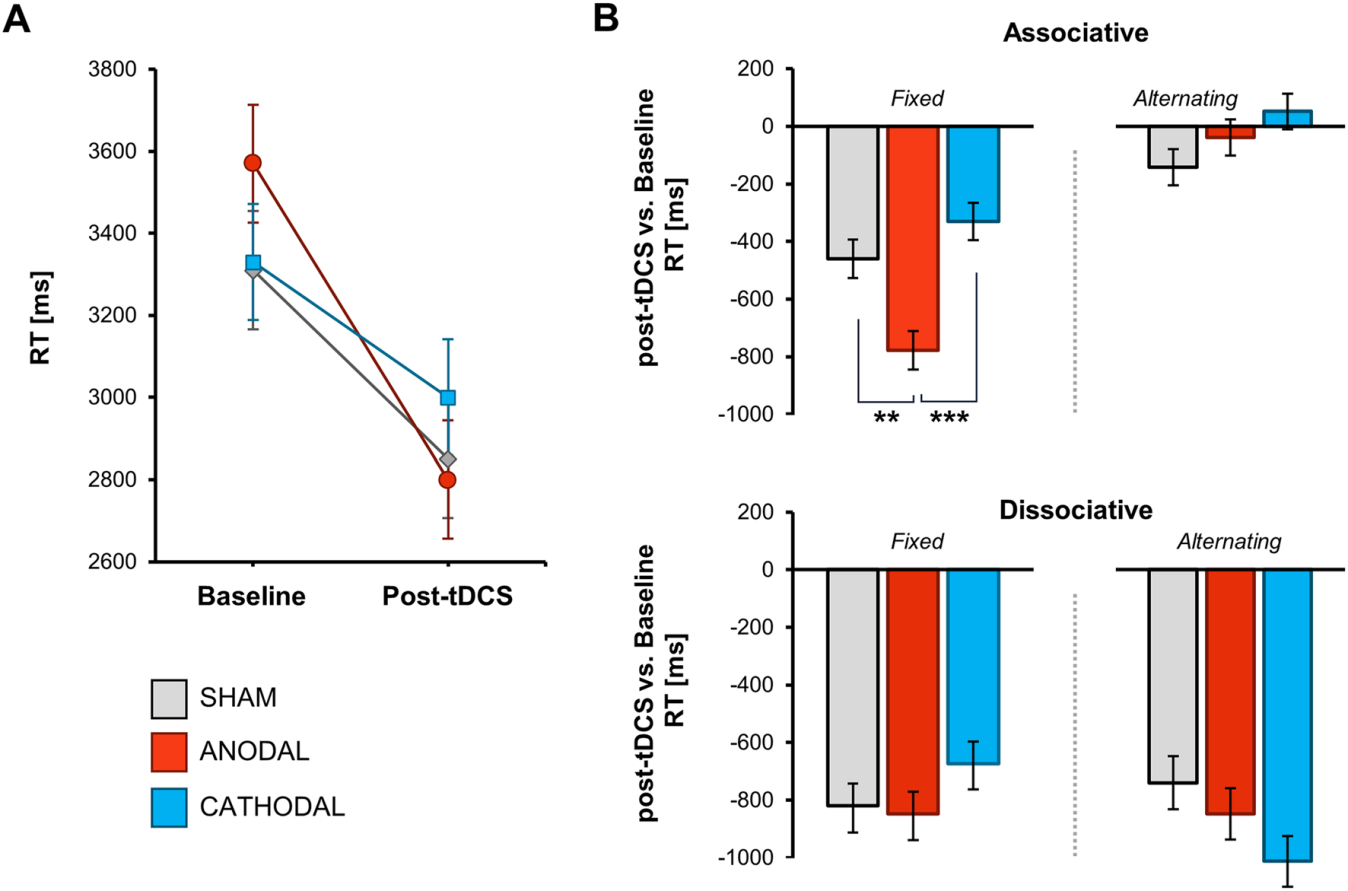
**A** The speed of word retrieval in the associated fixed condition of ACT. **B** The effect of cerebellar tDCS on performance in all ACT conditions. Plotted are difference RT (post-tDCS minus baseline) in each group estimated from the LMEM. Error bars: ± *SE*; Holm adjusted *p*-values: *p < .05, **p < .01, ***p < .001

The analysis showed no significant differences in performance between the experimental groups at baseline in any of the tasks: ACT, SCT, or CRTT (all *p* > 0.05, for more details please refer to Supplementary Table 1, Supplementary Figs. 1–7, and Fig. 2A). The different task conditions had the expected effect on the performance in all tasks (Fig. 1C). In ACT, in accordance with our previous studies (Marko et al. 2019, 2021, 2023), delivering semantically unrelated words (dissociations) required longer time than delivering related words (associations), and switching from associative to dissociative production further increased response latencies. In SCT, finishing unpredictable sentences took longer than finishing unpredictable sentences, and in CRTT, responses were longer in incongruent trials compared with congruent trials.

### The effects of cerebellar tDCS on ACT

The full-factorial LMEM indicated a significant interaction of *stimulation, block* and *condition*, *F*(6,20,575.3) = 3.45, *p* = 0.002, suggesting that the cerebellar tDCS affected the ACT performance in a specific way (a complete report of the LMEM results is provided in Supplementary Table 2). To disclose this complex effect, we separately analyzed each ACT condition (Table 2). A significant effect of *block* was present in all but Associative Alternating condition, reflecting that responses were overall faster in the second block. Importantly, in the Associative Fixed condition, a significant *block x* *stimulation* interaction was revealed (*F*(2, 5052.1) = 12.30, *p* < 0.001, Fig. 2A). To analyze the effects of stimulation in more detail, post-test minus baseline differences (ΔRT) were estimated from the LMEM and compared across groups. As depicted in Fig. 2B, the acceleration in fluency was significantly higher following anodal stimulation as compared with sham (group difference in ΔRT = − 318 ms, 95% CI [− 553, − 84], *p* = 0.001) and cathodal tDCS (group difference in ΔRT = − 449 ms, 95% CI [− 680, − 217], *p* < 0.001; also see Supplementary Table 3). The effect of cathodal tDCS was in the opposite direction than that of anodal stimulation, but the difference to sham stimulation was not significant (group difference in ΔRT =  + 130 ms, 95% CI [− 102, + 363], *p* = 0.161). The interaction between *block* and *stimulation* was not significant in any other ACT condition (Table 2, Supplementary Figs. 1–3).

**Table 2:** Statistical analysis of the experimental effects on ACT performance

Condition	Effect	*df1, df2*	*F*	*p*
Associative fixed	Stimulation	2, 132.6	0.17	.844
	Block	1, 5052.2	186.77	< .001***
	Block x Stimulation	2, 5052.1	12.30	< .001***
Associative alternating	Stimulation	2, 133.0	0.05	.951
	Block	1, 5124.4	1.38	.240
	Block x Stimulation	2, 5124.4	2.42	.089
Dissociative fixed	Stimulation	2, 133.2	0.74	.480
	Block	1, 5060.1	302.68	< .001***
	Block x Stimulation	2, 5060.1	1.48	.228
Dissociative alternating	Stimulation	2, 132.8	0.62	.541
	Block	1, 4949.3	279.61	< .001***
	Block x Stimulation	2, 4949.3	2.35	.096

*p < .05, **p < .01, ***p < *.001* (after Tukey HSD adjustment)

### The effects of cerebellar tDCS on SCT

The analysis showed significant effects of *block, F*(1, 15,554) = 39.0, *p* < 0.001, and *condition, F*(1, 15,554.1) = 13,963.8, *p* < 0.001, reflecting overall faster responses during the post-tDCS vs. baseline block (ΔRT = − 3 ms, 95% CI [− 69, – 36], *p* < 0.001), and in predictable vs. unpredictable sentences (ΔRT = − 998 ms, 95% CI [−1001, − 982], *p* < 0.001). However, these effects were independent of *stimulation* (*block* x *condition* x *stimulation, F*(2, 15,553.9) = 1.21, *p* = 0.298), indicating that tDCS had no effect on the latency of sentence completion. For complete results of the analysis and depiction of the experimental effects please refer to Supplementary Tables 4–6 and Supplementary Figs. 4–5.

### The effects of cerebellar tDCS on CRTT

The effects of *block*, *F*(1, 63,322) = 6381.78, *p* < 0.001, and *condition*, *F*(1, 63,321) = 32,980.16, *p* < 0.001, were significant, reflecting shorter RTs in the post-tDCS vs. baseline block, and in congruent vs. incongruent trials. Furthermore, there was a significant interaction between *stimulation, block,* and *condition*, *F*(2, 63,320) = 5.95, *p* = 0.003. Separate analyses for the two task conditions revealed a significant *block x stimulation* interaction in congruent trials, *F*(2, 47,766) = 7.33, *p* < 0.001. To analyze the effects of stimulation in more detail, post-test minus baseline differences (ΔRT) were estimated from the LMEM and compared across groups. While all three groups had shorter RTs in the post-tDCS block vs. baseline, when compared with the sham group, this improvement in RT was significant only in the anodal group (group difference in ΔRT = − 5 ms, 95% CI [– 9, 1], *p* = 0.019), but not in the cathodal group (group difference in ΔRT =  + 2 ms, 95% CI [– 2 + 6], *p* = 0.255), and the anodal and the cathodal group also differed significantly (group difference in ΔRT = − 7 ms, 95% CI [– 11, − 2], *p* < 0.001). For incongruent trials, the *block x stimulation* interaction was not significant, *F*(2, 15,426) = 2.07, *p* = 0.126. For complete results of the analysis and depiction of the experimental effects please refer to Supplementary Tabs. 7–9 and Supplementary Figs. 6–7.

## Discussion

This study used tDCS and ACT to assess the contribution of the cerebellum to automatic (free-associative) versus controlled (inhibition, switching) semantic memory retrieval, respectively. We revealed that anodal cerebellar tDCS facilitated the retrieval of sequentially related concepts within the free-associative word chains but had no influence on the retrieval conditions that pose demands on semantic control, i.e., inhibition (delivery of unrelated words) or switching (flexible alternating between the retrieval rules). Our data thus indicate that the cerebellum is engaged in automatic rather than controlled retrieval from semantic memory. Furthermore, anodal cerebellar stimulation inhibited rather than facilitated response latencies in the simple choice reaction time task. Although the behavioral effects in this basic task were very small (but statistically significant), they rule out that the improved associative fluency was due to a non-specific (domain-general) acceleration in processing speed.

There is abundant evidence that the cerebellum supports automatic processing and habit formation, which has been attributed to the so-called forward model of control implemented within the cortico-cerebellar neuronal circuitry (Wolpert et al. 1998; Ito 2008). In this type of control, the command signal (which drives the effector) is adaptively adjusted to minimize an error signal that originates from a discrepancy between the outcome (transmitted by a feedback signal) and the outcome’s prediction (generated based on an efference copy of the command signal). Through repetition and learning, the efficiency of the process execution gradually increases so that needs for ongoing cognitive control decrease and the process becomes automated. In line with this perspective, numerous research studies have shown that anodal cerebellar tDCS facilitates motor adaptation, learning and retention of the learned motor skills, the hallmark of which is automation (e.g., Galea et al. 2011; Kumari et al. 2020; Yavari et al. 2016; for review see Kumari et al. 2019; Tzvi et al. 2022, but see Jalali et al. 2017). It has been proposed that the forward model, which employs cerebellar plasticity, reach beyond motor control and also mediates automation of cognitive processes (Houk 1997; Ramnani 2014; Shine and Shine 2014; Koziol et al. 2014). Relatedly, in the domain of language processing, number of studies have documented the role of the cerebellum in language development and speech production (for review see e.g., Ackermann and Brendel 2016; Mariën et al. 2014; Mariën and Borgatti 2018; Ziegler 2016). Furthermore, the cerebellum is active, and cerebellar patients are impaired, in word generation paradigms such as verbal fluency tasks (Molinari and Leggio 2016; Mariën and Borgatti 2018). However, since verbal fluency is a hybrid measure employing automatic as well as controlled semantic processing (Shao et al. 2014; Michalko et al. 2023; Marko et al. 2023), the previous evidence remains inconclusive on whether the cerebellum contributes to either or both. Furthermore, the reports of the effects of cerebellar tDCS on verbal fluency are inconsistent (Turkeltaub et al. 2016; Bongaerts et al. 2022). On the other hand, generating free associates is considered as a relatively pure measure of automatic (i.e., spontaneous and unconstrained) memory retrieval with little demands on executive control processes (Marron et al. 2018; Gray et al. 2019; Marko et al. 2019). The present findings thus indicate that the cerebellum supports the automatic lexical-semantic retrieval, which is driven by the established (habitual) structure of semantic representations.

The effects of cerebellar stimulation on retrieving semantically related information may not be general, since the associative fluency was improved in the fixed but not the alternating chains. This difference may be due to the distinct feature of the alternating associative-dissociative task, in which each attempt to retrieve a semantically related word is preceded by a switch and therefore discontinued. Consequently, delivering associative responses in alternating chains represents isolated events, diminishing the benefit from ongoing consecutive pre-activation (i.e., forward activation) of the related representations within memory, afforded by the fixed chains. Furthermore, the cerebellar tDCS did not affect the latency of completing sentences, which may also exert demands on predictive semantic processing and conceptual access. Although this result apparently differs from some previous studies reporting positive effects of anodal cerebellar tDCS on the speed of sentence completion (D’Mello et al. 2017; Rice et al. 2021), the SCT paradigm employed in those studies involved a two-alternative choice of the final word, in contrast to a free word recall used in our experiment. Also, since the cues in SCT were presented for 500 ms each, it is possible that participants in our study had been able to predict the sentence ending before being probed to respond, hence producing a ceiling effect that could eventually mask any benefits on RT from the cerebellar stimulation. Hence, the task features and parameters may moderate the effects of cerebellar tDCS on sentence completion ability, warranting further research in this direction. Finally, while SCT involves semantic integration of multiple cues to converge on a single likely response, the free–associative task represents an unconstrained, diverging mode where single cue probes multiple distributed memory representations. These processing differences may eventually account for a distinct effect of cerebellar tDCS on open-ended free-associative production (employed in ACT) and sentence completion.

The three groups in our study showed no statistically significant differences in baseline performance. Nevertheless, before stimulation, the fluency in the Associate Fixed condition of ACT was slowest in the anodal group, potentially raising question about the interpretation of the significant interaction between *block* and *stimulation* in this condition. On the one hand, it has been reported that individuals with lower performance or stronger functional impairment could be more sensitive to neurostimulation intervention (e.g. Krebs et al. 2021). On the other hand, apart from the fact that the group differences in our study were not significant, the performance in the anodal group became best of all groups following the stimulation and this group showed improvement in fluency that was bigger than the group differences at baseline. It is thus improbable that the results were distorted by baseline group differences. Since our study included healthy individuals it is premature to speculate about potential implications of our findings for clinical treatment or rehabilitation. Yet, to identify the individuals who best benefit from the application of neurostimulation methods is undoubtedly one important goal for future research in this field in general.

One matter of debate regarding the cerebellum is the lateralization of cerebellar functions. Although neuroimaging studies employing language tasks usually report stronger activation of the right than the left cerebellum, meta-analyses have confirmed the activations in both the right and the left cerebellar hemispheres (Stoodley and Schmahmann 2009; Keren-Happuch et al. 2014). Similarly, findings from patients affected by cerebellar pathologies show that the language processing in the cerebellum is not unilateral. In particular, impairments in verbal fluency may result from both right or left cerebellar lesions (Molinari and Leggio 2016). Moreover, recent meta-analysis of transcranial magnetic stimulation studies by Gatti et al. (2021b) has not confirmed that the laterality of cerebellar stimulation critically drives the stimulation-induced effects on cognition. Since we adopted bilateral stimulation of the cerebellum, the question remains open whether the observed effects of tDCS were due to the modulation of both or just one of the hemispheres.

Notably, the results of the current study complement those from our previous experiment, which has shown that anodal tDCS of the left lateral prefrontal cortex facilitated dissociative performance in ACT but had no influence on retrieval of semantic associates, thus boosting controlled but not automatic semantic retrieval (Marko and Riečanský 2021). These empirical findings fit well into the conceptual framework proposed by Ramnani (2014) who suggested that automatic (fast, effortless) processing across the motor and the cognitive domains is supported by the cerebellum, while controlled (slow, effortful) processing relies on the computations within the frontal cortex. Our data provide support for the engagement of the cerebellum in automation of cognitive function. The fact that the excitatory cerebellar tDCS speeded up specifically the continuous free-associative retrieval suggests that the cerebellum may contribute to automatic memory retrieval by facilitating forward propagation of semantic activation patterns across the conceptual representations acquired and strengthened (automated) by experience (i.e., learning). This mechanism may underpin the predictive role of the cerebellum in language and high-level cognition.

### Supplementary Information

Below is the link to the electronic supplementary material.

Supplementary file1 (PDF 1106 KB)

## Data Availability

Additional information about the methods and results of the study is provided in Supplementary material online. Data from this study are openly available in OSF at https://doi.org/10.17605/OSF.IO/VF8HS.
