# Supplementary material for: Electrical stimulation of the cerebellum facilitates automatic but not controlled word retrieval
Source: Brain Struct Funct. 2023 Oct 2;228(9):2137–46. doi: 10.1007/s00429-023-02712-0 (PMC10587269; doi:10.1007/s00429-023-02712-0)
Supplement: Supplementary file 1 — Supplementary file1 (PDF 1106 KB) [file 429_2023_2712_MOESM1_ESM.pdf]

Supplementary information

Electrical stimulation of the cerebellum facilitates automatic but not controlled word retrieval

Petrikova, Marko, Rovny & Riecansky (2023)

Contents

Sentence completion task – the pilot study 1
Side effects of tDCS 2
Tab. 1. Statistical tests of group differences (factor Stimulation) in task performance at baseline (pre-tDCS) 3
Tab. 2. ACT: analysis of the experimental effects 3
Tab. 3. ACT: pairwise group comparisons of ΔRT (post-tDCS vs. baseline) 4
Tab. 4. SCT: analysis of the experimental effects 4
Tab. 5. SCT: analysis of the experimental effects in each condition 5
Tab. 6. SCT: pairwise group comparisons of ΔRT (post-tDCS vs. baseline) 5
Tab. 7. CRTT: analysis of the experimental effects 6
Tab. 8. CRTT: analysis of the experimental effects in each condition 6
Tab. 9. CRTT: pairwise group comparisons of ΔRT (post-tDCS vs. baseline) 7
Tab. 10. Ratings of the sensations elicited by tCDS 7
Tab 11. Association between stimulation-induced sensations and ΔRT in ACT performance 8
Fig. 1: ACT: the effect of tDCS in the Associative Alternating condition 9
Fig. 2. ACT: the effect of tDCS in the Dissociative Fixed condition 10
Fig. 3. ACT: the effect of tDCS in the Dissociative Alternating condition 11
Fig. 4. SCT: the effect of tDCS on completion of predictable sentences 12
Fig. 5. SCT: the effect of tDCS on completion of unpredictable sentences 13
Fig. 6: CRTT: the effect of tDCS on response latencies in congruent trials 14
Fig. 7. CRTT: the effect of tDCS on response latency in incongruent trials 15

Abbreviations:

ACT – associative chain task, LMEM – linear mixed effect model, CRTT – choice reaction time task, RT – response time/latency, SCT – sentence completion task, tDCS – transcranial direct current stimulation

Sentence completion task – the pilot study

Prior to the experiment, the set of 140 generated unfinished sentences was administered to 20 subjects (not participating in the subsequent experiment) who were instructed to type the

finishing words so the cloze probability of sentences could be checked. Evaluation criteria were in line with Block and Baldwin (Block & Baldwin, 2010) and were as follows: words with one missing/additional/transposed letter were included and counted as the intended word; synonyms and similar words were not counted toward the intended word (i.e., words "gun" and "rifle" were counted as separate responses). Based on the cloze probability scores 60 predictable and 60 unpredictable sentences were selected from the list to be used in this experiment.

Side effects of tDCS

The side effects of stimulation were measured with a modified and translated version of a sensation questionnaire used by Fertonani et al. (2010). In this questionnaire, various possible discomforting adverse effects were described (itchiness, pain, burning, heat, pinching, iron taste or fatigue) and participants were asked to score them on a scale from 1 (no sensation) to 10 (very strong sensation). Besides that, the questionnaire contained questions to determine when the sensations began, how long they lasted and also participant's assessment whether the active or sham stimulation was delivered.

The stimulation was well tolerated. The mean intensities of sensations reported are shown in Tab. 10. Given that the data for each reported sensation were not normally distributed (Shapiro-Wilk tests yielded all $p < .05$), a nonparametric Kruskal-Wallis test was carried out to compare the groups. Vast majority of the participants reported the sensations only in the beginning of the stimulation (presumably during ramp-up). The guess of the applied stimulation (real vs. sham) was at chance level (55.1% correct).

Associations between the sensations and stimulation effects in ACT are shown in Tab. 11. Notably, ratings of itching were unrelated to difference RT (post-tDCS minus baseline) and thus do not explain the effects of stimulation (group differences) in this condition.

Tab. 1. Statistical tests of group differences (factor Stimulation) in task performance at baseline (pre-tDCS)

Task	***Condition***	***df1, df2***	***F***	***p***
**ACT**	Associative Fixed	2, 131.21	1.03	.360
	Associative Alternating	2, 132.78	0.34	.709
	Dissociative Fixed	2, 131.82	0.56	.573
	Dissociative Alternating	2, 131.96	0.42	.658
**SCT**	Predictable sentences	2, 132.93	0.13	.874
	Unpredictable sentences	2, 135.68	0.86	.427
**CRTT**	Congruent trials	2, 131.98	0.81	.447
	Incongruent trials	2, 130.97	1.03	.359

RTs were analyzed using LMEM (Tukey HSD adjusted *p*-values are reported).

Tab. 2. ACT: analysis of the experimental effects

Factor	***df1, df2***	***F***	***p***
Stimulation	2, 132.9	0.32	.724
Block	1, 20575.3	542.84	$< .001^{***}$
Condition	3, 20577.1	1947.99	$< .001^{***}$
Stimulation x Block	2, 20575.3	2.94	.053
Stimulation x Condition	6, 20577.1	11.41	$< .001^{***}$
Block x Condition	3, 20575.3	61.90	$< .001^{***}$
Stimulation x Block x Condition	6, 20575.3	3.45	$.002^{**}$

RTs were analyzed using LMEM. $^{*}p < .05$, $^{**}p < .01$, $^{***}p < .001$ (Tukey HSD adjusted)

Tab. 3. ACT: pairwise group comparisons of ΔRT (post-tDCS vs. baseline)

Condition	*Contrast*	*Difference in ms (±SE)*	*p*
**Associative Fixed**	A vs S	-318.0 (±94)	.001**
	C vs S	+130.0 (±93)	.161
	A vs C	-449.0 (±93)	< .001***
**Associative Alternating**	A vs S	+103.2 (±89)	.733
	C vs S	+193.3 (±88)	.111
	A vs C	-90.1 (±88)	.733
**Dissociative Fixed**	A vs S	-28.3 (±111)	.799
	C vs S	+146.5 (±110)	.547
	A vs C	-174.7 (±109)	.440
**Dissociative Alternating**	A vs S	-107.8 (±128)	.801
	C vs S	-272.6 (±127)	.128
	A vs C	+164.8 (±126)	.569

The values were estimated from LMEM. Abbreviations: A (anodal group), C (cathodal group), S (sham group). $^{*}p < .05$, $^{**}p < .01$, $^{***}p < .001$ (Holm adjusted)

Tab. 4. SCT: analysis of the experimental effects

Factor	*Df1, df2*	*F*	*p*
Stimulation	2, 136.8	0.63	.532
Block	1, 15554.0	39.00	< .001***
Condition	1, 15554.1	13963.82	< .001***
Stimulation x Block	2, 15554.0	0.11	.894
Stimulation x Condition	2, 15554.1	12.15	< .001***
Block x Condition	1, 15553.9	15.25	< .001***
Stimulation x Block x Condition	2, 15553.9	1.21	.298

RTs were analyzed using LMEM. $^{*}p < .05$, $^{**}p < .01$, $^{***}p < .001$ (Tukey HSD adjusted)

Tab. 5. SCT: analysis of the experimental effects in each condition

Condition	*Effect*	*Df1, df2*	*F*	*p*
**Predictable sentences**	*Stimulation*	2, 132.9	0.30	.738
	Block	1, 7715.0	13.03	$< .001^{***}$
	Block x Stimulation	2, 7715.0	1.69	.185
**Unpredictable sentences**	*Stimulation*	2, 138.0	0.80	.453
	Block	1, 7706.0	32.43	$< .001^{***}$
	Block x Stimulation	2, 7706.0	0.67	.513

RTs were analyzed using LMEM. $^{*}p < .05$, $^{**}p < .01$, $^{***}p < .001$ (Tukey HSD adjusted)

Tab. 6. SCT: pairwise group comparisons of ΔRT (post-tDCS vs. baseline)

Condition	*Contrast*	*Difference in ms (±SE)*	*p*
**Predictable sentences**	A vs S	+8 (±10)	.535
	C vs S	-16 (±10)	.487
	A vs C	+24 (±10)	.284
**Unpredictable sentences**	A vs S	-3 (±40)	1.000
	C vs S	+35 (±40)	1.000
	A vs C	-38 (±40)	1.000

The values estimated from LMEM. Abbreviations: A (anodal group), C (cathodal group), S (sham group) (Holm adjusted *p*-values are reported)

Tab. 7. CRTT: analysis of the experimental effects

Factor	*df1, df2*	*F*	*p*
Stimulation	2, 133.0	1.65	.195
Block	1, 63322.0	6381.78	< .001***
Condition	1, 63321.0	32980.16	< .001***
Stimulation x Block	2, 63322.0	0.49	.610
Stimulation x Condition	2, 63321.0	25.03	< .001***
Block x Condition	1, 63320.0	53.52	< .001***
Stimulation x Block x Condition	2, 63320.0	5.95	.003**

RTs were analyzed using LMEM. *p < .05, **p < .01, ***p < .001 (Tukey HSD adjusted)

Tab. 8. CRTT: analysis of the experimental effects in each condition

Condition	*Effect*	*Df1, df2*	*F*	*p*
**Congruent trials**	*Stimulation*	2, 133.0	1.17	.313
	Block	1, 47766.0	6059.65	< .001***
	Block x Stimulation	2, 47766.0	7.33	< .001***
**Incongruent trials**	*Stimulation*	2, 132.0	1.30	.275
	Block	1, 15426.0	2315.62	< .001***
	Block x Stimulation	2, 15426.0	2.07	.126

RTs were analyzed using LMEM. *p < .05, **p < .01, ***p<.001 (after Tukey HSD adjustment)

Tab. 9. CRTT: pairwise group comparisons of ΔRT (post-tDCS vs. baseline)

Condition	*Contrast*	*Difference in ms (±SE)*	*p*
**Congruent trials**	A vs S	+4.6 (±1.8)	.019*
	C vs S	-2.0 (±1.8)	.255
	A vs C	+6.6 (±1.8)	< .001***
**Incongruent trials**	A vs S	-6.7 (±3.5)	.187
	C vs S	-1.6 (±3.5)	.651
	A vs C	-5.1 (±3.4)	.267

Values were estimated from LMEM. Abbreviations: A (anodal group), C (cathodal group), S (sham group). $^{*}p < .05$, $^{**}p < .01$, $^{***}p < .001$ (Holm adjusted)

Tab. 10. Ratings of the sensations elicited by tCDS

	**Stimulation**			
	Anodal	***Cathodal***	***Sham***	***Statistical analysis***
Itching	6.31 (±2.85)	4.36 (±3.35)	4.02 (±2.80)	$\chi^2(2) = 13.68$, $p = .001^{**}$
Pain	3.24 (±2.51)	2.33 (±2.11)	2.68 (±2.43)	$\chi^2(2) = 5.32$, $p = .070$
Burning	4.93 (±2.78)	4.89 (±3.03)	3.96 (±2.86)	$\chi^2(2) = 3.25$, $p = .197$
Heat	3.71 (±2.78)	3.61 (±2.77)	2.43 (±2.19)	$\chi^2(2) = 6.86$, $p = .032^{*}$
Tingling	4.82 (±2.73)	4.59 (±3.24)	3.98 (±3.16)	$\chi^2(2) = 2.14$, $p = .344$
Iron taste	1.09 (±0.29)	1.47 (±1.22)	1.18 (±0.91)	$\chi^2(2) = 5.98$, $p = .050$
Fatigue	3.02 (±2.43)	4.24 (±3.26)	3.34 (±2.53)	$\chi^2(2) = 3.56$, $p = .168$

Data represent mean (±SD). The intensity was reported on a 10-point scale ranging from 1 to 10. Data were analyzed using Kruskal-Wallis test. $^{*}p < .05$, $^{**}p < .01$

Tab 11. Association between stimulation-induced sensations and ΔRT in ACT performance

	ΔRT			
	AF	***AA***	***DF***	***DA***
Itching	-0.014	0.063	0.034	0.023
Pain	-0.094	-0.173	-0.078	-0.086
Burning	-0.041	0.011	-0.040	-0.086
Heat	-0.204	-0.126	-0.089	-0.138
Tingling	0.008	0.007	0.093	-0.018
Iron taste	0.066	0.023	-0.066	-0.001
Fatigue	0.057	-0.074	0.007	-0.021

Data: values of Pearson's correlation coefficient (n = 136), ΔRT: post-tDCS minus baseline RT differences estimated from LMEM, AF: associate fixed, AS: associate alternating, DF: dissociate fixed, DA: dissociate alternating. No correlation was statistically significant (p-values were adjusted using Holm's correction).

Fig. 1: ACT: the effect of tDCS in the Associative Alternating condition

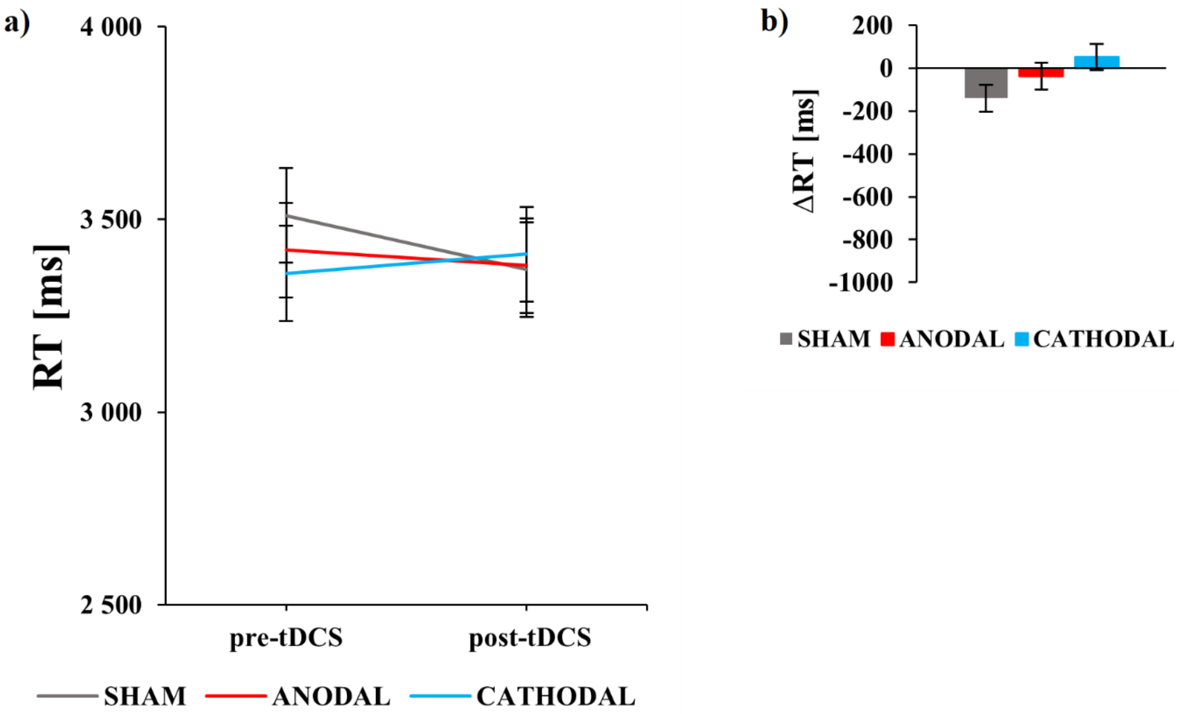

a) Mean response time (RT) for each experimental group before (baseline, pre-tDCS) and after stimulation (post-tDCS); **b)** Mean difference RT (post-tDCS vs. baseline) in each group estimated from LMEM. Error bars represent ± *SE*.

Fig. 2. ACT: the effect of tDCS in the Dissociative Fixed condition

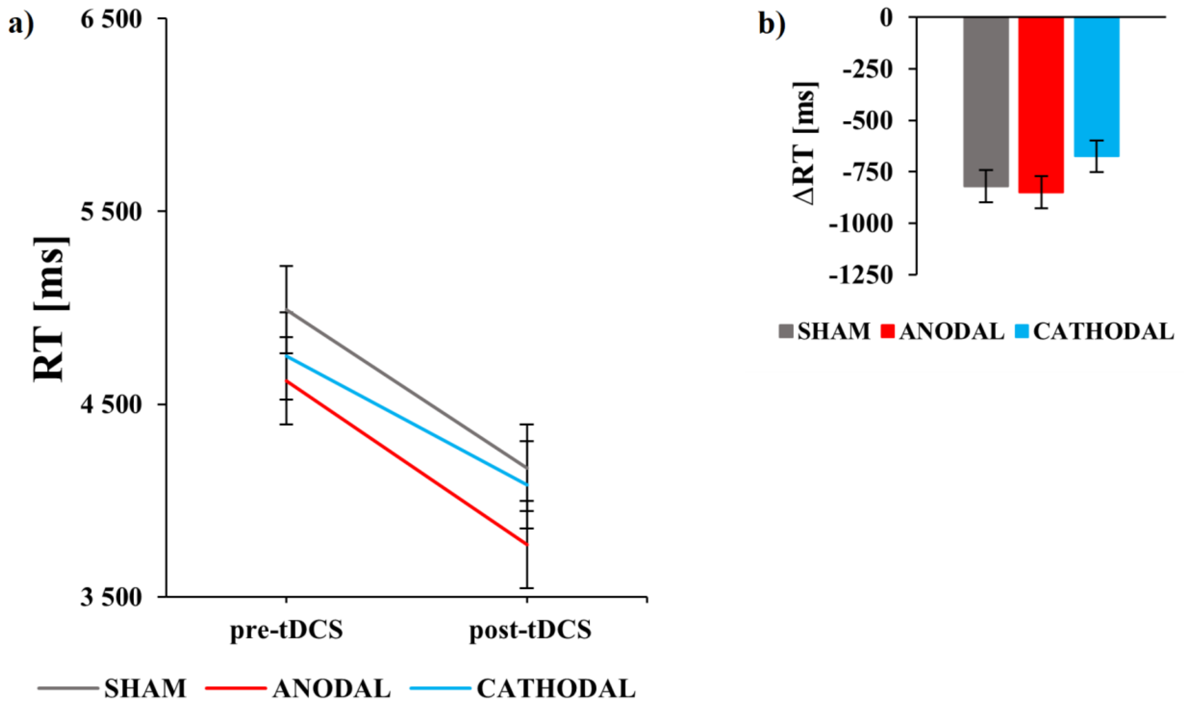

a) Mean response time (RT) for each experimental group before (baseline, pre-tDCS) and after stimulation (post-tDCS); **b)** Mean difference RT (post-tDCS vs. baseline) in each group estimated from LMEM. Error bars represent ± *SE*.

Fig. 3. ACT: the effect of tDCS in the Dissociative Alternating condition

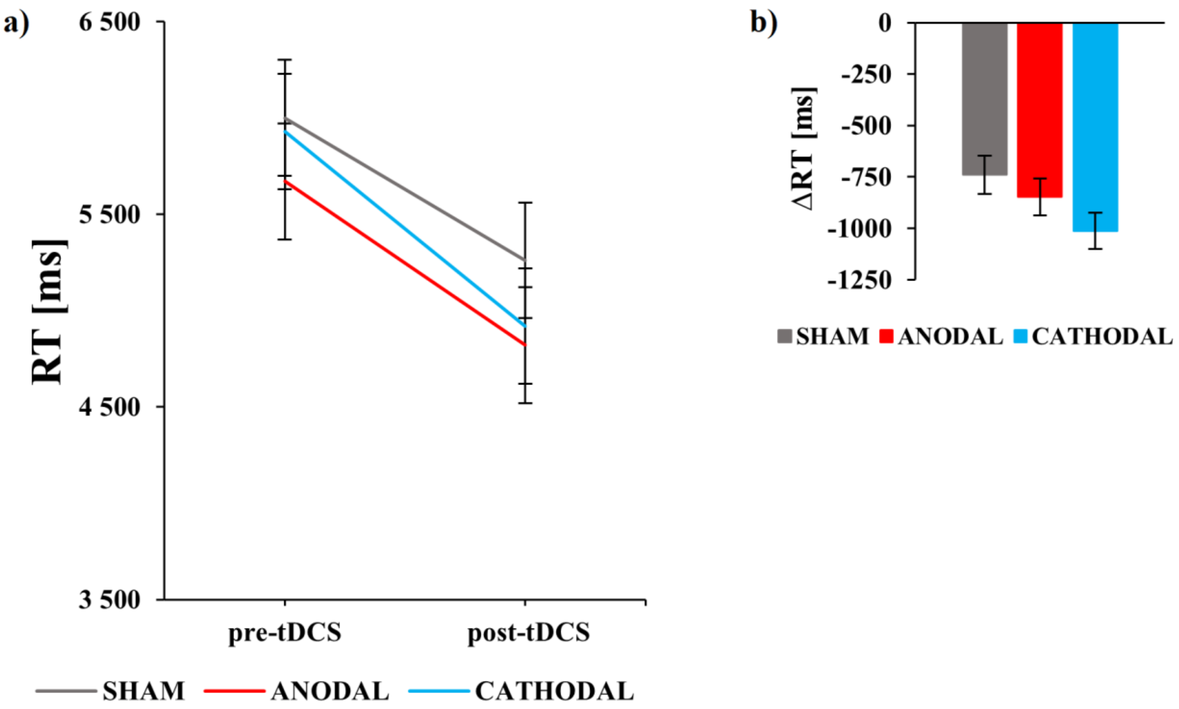

a) Mean response time (RT) for each experimental group before (baseline, pre-tDCS) and after stimulation (post-tDCS); **b)** Mean difference RT (post-tDCS vs. baseline) in each group estimated from LMEM. Error bars represent ± *SE*.

Fig. 4. SCT: the effect of tDCS on completion of predictable sentences

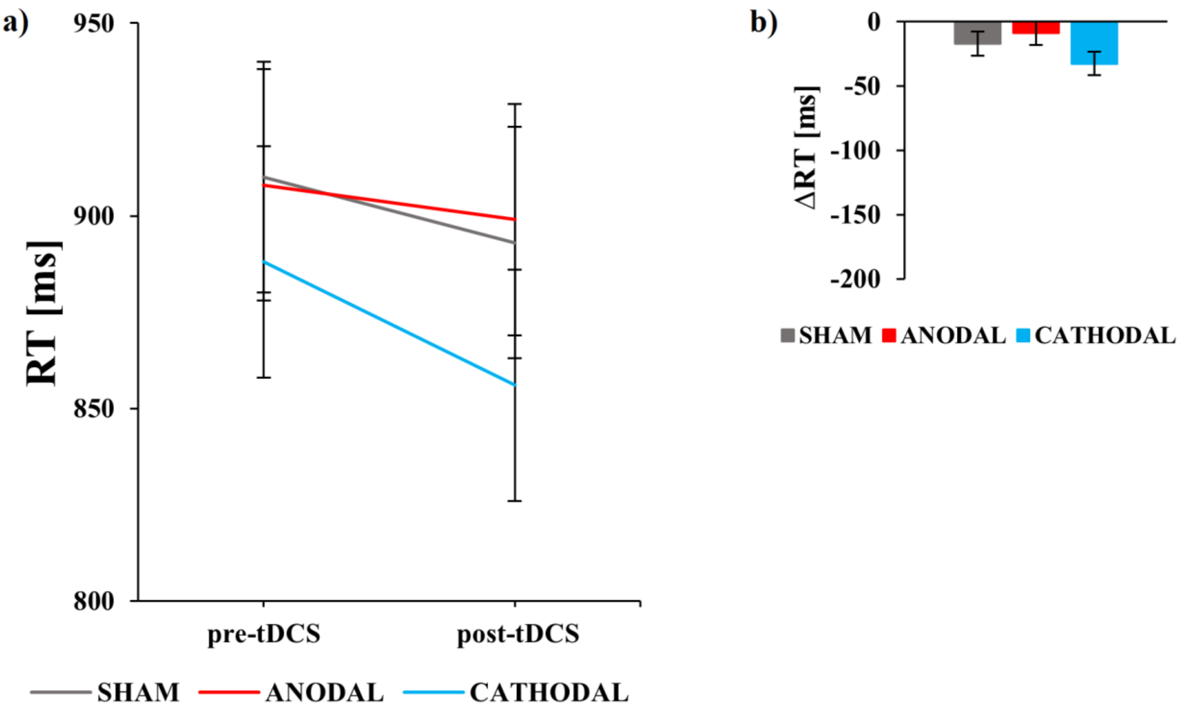

a) Mean response time (RT) for each experimental group before (baseline, pre-tDCS) and after stimulation (post-tDCS). **b**) Mean difference RT (post-tDCS vs. baseline) in each group estimated from LMEM. Error bars represent ± *SE*.

Fig. 5. SCT: the effect of tDCS on completion of unpredictable sentences

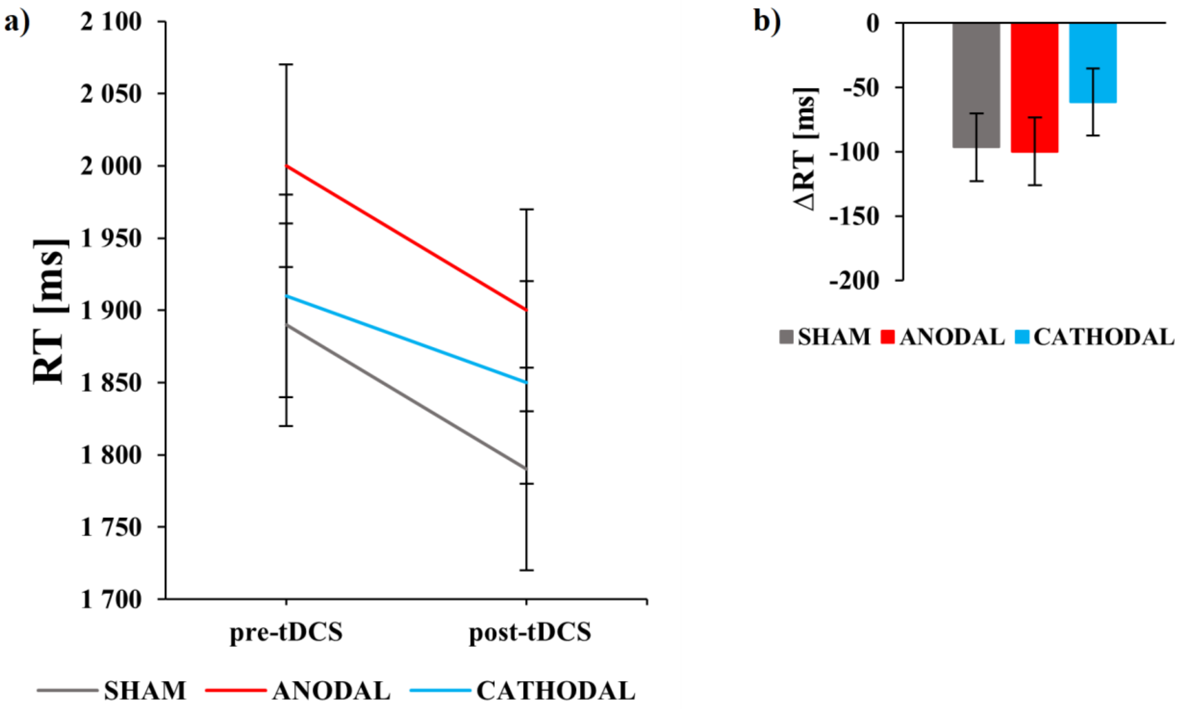

a) Mean response time (RT) for each experimental group before (baseline, pre-tDCS) and after stimulation (post-tDCS). **b**) Mean difference RT (post-tDCS vs. baseline) in each group estimated from LMEM. Error bars represent ± *SE*.

Fig. 6: CRTT: the effect of tDCS on response latencies in congruent trials

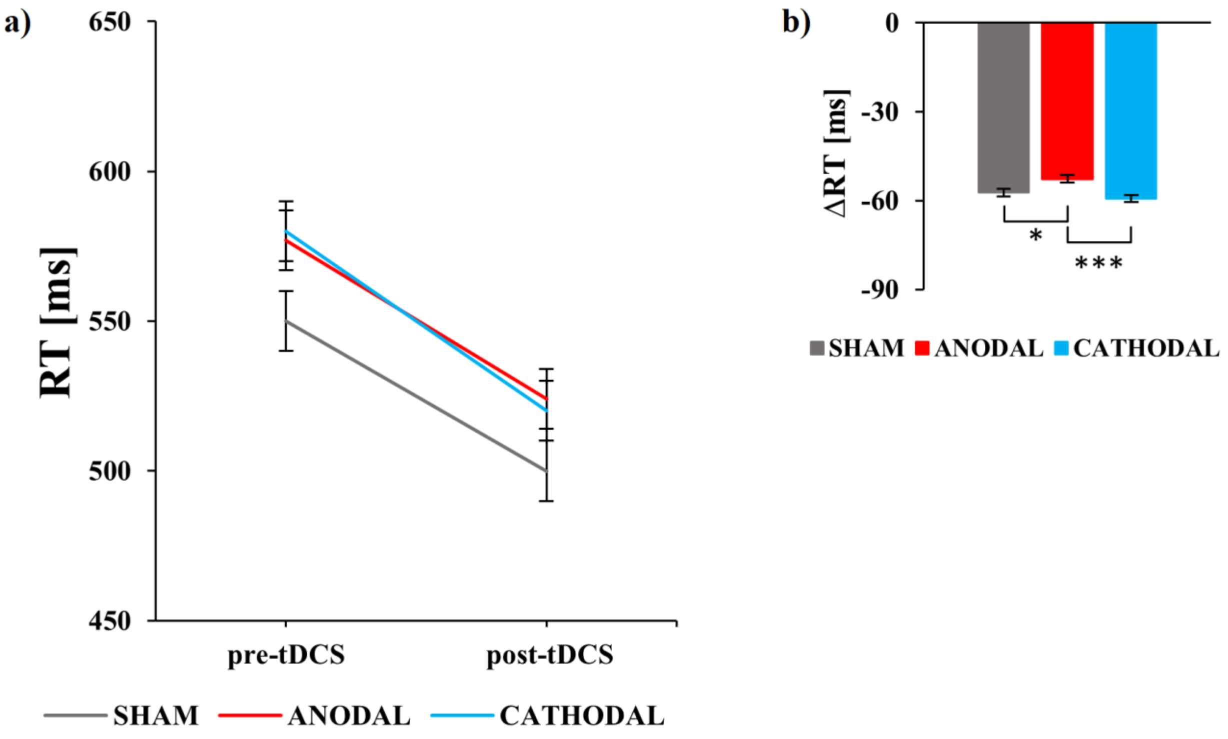

a) Mean response times (RT) for all experimental groups before (baseline, pre-tDCS) and after stimulation (post-tDCS); **b)** Mean difference RT (post-tDCS vs. baseline) in each group estimated from LMEM. Error bars represent ± *SE*; Holm adjusted *p*-values are reported. $^{*}p < .05$, $^{**}p < .01$, $^{***}p < .001$

Fig. 7. CRTT: the effect of tDCS on response latency in incongruent trials

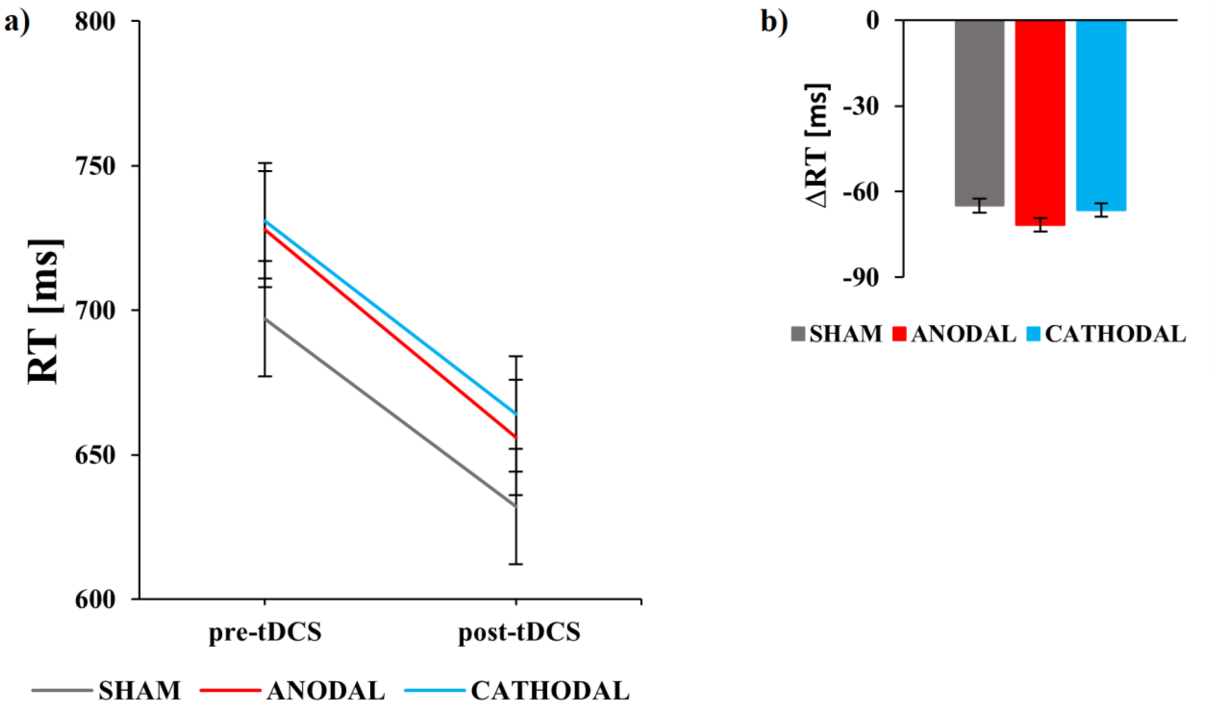

a) Mean response time (RT) for each experimental group before (baseline, pre-tDCS) and after stimulation (post-tDCS). **b**) Mean difference RT (post-tDCS vs. baseline) in each group estimated from LMEM. Error bars represent ± *SE*.

References

Block, C. K., & Baldwin, C. L. (2010). Cloze probability and completion norms for 498 sentences: Behavioral and neural validation using event-related potentials. *Behavior Research Methods*, *42*(3), 665–670. https://doi.org/10.3758/BRM.42.3.665

Fertonani, A., Rosini, S., Cotelli, M., Rossini, P. M., & Miniussi, C. (2010). Naming facilitation induced by transcranial direct current stimulation. *Behavioural Brain Research*, *208*(2), 311–318. https://doi.org/10.1016/j.bbr.2009.10.030
